# Spectral Profiling of Early αsyn Aggregation in HEK293 Cells Modified to Stably Express Human WT and A53T-αsyn

**DOI:** 10.3390/cells14191542

**Published:** 2025-10-02

**Authors:** Priyanka Swaminathan, Karsten Sættem Godø, Eline Bærøe Bjørn, Therése Klingstedt, Debdeep Chatterjee, Per Hammarström, Rajeevkumar Raveendran Nair, Mikael Lindgren

**Affiliations:** 1 Department of Physics, Faculty of Natural Sciences, Norwegian University of Science and Technology (NTNU), NO-7491 Trondheim, Norway; 2 Department of Physics, Chemistry and Biology, Linköping University, SE-581 83 Linköping, Swedenper.hammarstrom@liu.se (P.H.); 3 SciLifeLab, Linköping University, SE-581 83 Linköping, Sweden; 4 Kavli Institute for Systems Neuroscience, Norwegian University of Science and Technology (NTNU), Olav Kyrres Gate 9, NO-7030 Trondheim, Norway; rajeevkumar.r.nair@ntnu.no

**Keywords:** alpha-synuclein, Parkinson’s disease, pre-formed fibrils, A53T mutation, fibrillation kinetics assay, ThT, h-FTAA, stable αsyn, HEK293 cells, spectral imaging, fluorescence lifetime imaging

## Abstract

Alpha-synuclein (αsyn) misfolding and aggregation underlie several neurodegenerative disorders, including Parkinson’s disease. Early oligomeric intermediates are particularly toxic yet remain challenging to detect and characterize within cellular systems. Here, we employed the luminescent conjugated oligothiophene h-FTAA to investigate early aggregation events of human wildtype (huWT) and A53T-mutated αsyn (huA53T) both in vitro and in HEK293 cells stably expressing native human-αsyn. Comparative fibrillation assays revealed that h-FTAA detected αsyn aggregation with higher sensitivity and earlier onset than Thioflavin T, with the A53T variant displaying accelerated fibrillation. HEK293 cells stably expressing huWT- or huA53T-αsyn were exposed to respective pre-formed fibrils (PFFs), assessed via immunocytochemistry, h-FTAA staining, spectral emission profiling, and fluorescence lifetime imaging microscopy (FLIM). Notably, huA53T PFFs promoted earlier aggregation patterns and yielded narrower fluorescence lifetime distributions compared with huWT PFFs. Spectral imaging showed h-FTAA emission maxima (~550–580 nm) red-shifted and broadened in cells along with variable lifetimes (0.68–0.87 ns), indicating heterogeneous aggregate conformations influenced by cellular milieu. These findings demonstrate that h-FTAA is useful for distinguishing early αsyn conformers in living systems and, together with stable αsyn-expressing HEK293 cells, offers a platform for probing early αsyn morphotypes. Taken together, this opens for further discovery of biomarkers and drugs that can interfere with αsyn aggregation.

## 1. Introduction

Protein aggregation, triggered by the misfolding and buildup of the pathogenic protein alpha-synuclein (αsyn), forms the pathological feature of Parkinson’s Disease (PD), Multiple System Atrophy (MSA), and Dementia with Lewy Bodies (DLB) [1]. These neurodegenerative disorders are collectively referred to as synucleinopathies. The disease-prone αsyn is thought to exist in multiple conformation states and morphologies, likely influencing the spectrum of related clinical and phenotypic manifestations [2,3,4]. Notably, the intermediate oligomeric, early-stage aggregate species of αsyn are suggested to impart elevated cytotoxicity compared to the matured αsyn conformers [5,6,7,8,9,10].

Moreover, several studies have suggested the potential influence of the cellular environment in modulating the morphology of αsyn conformers, influencing their aggregation behavior [11,12,13]. However, the caveat persists in detecting the early αsyn proteoforms and assessing the underlying molecular environments likely influencing the protein morphologies [14,15]. Assessment of early αsyn morphotypes could be useful in uncovering the molecular bases of synucleinopathies and finding potential biomarkers of early diagnosis. To this end, luminescent conjugated oligothiophenes (LCOs) have been widely used for detecting disease-associated protein aggregates, including amyloid polymorphs, by leveraging their conformation-sensitive fluorescence characteristics [16,17]. The flexible thiophene backbone offers adaptive binding to structurally diverse protein conformers, manifesting in distinct spectral patterns and fluorescence lifetimes reflective of the surrounding molecular environment [17,18]. The pentameric thiophene derivative, pentamer formyl thiophene acetic acid (p-FTAA), has been shown to bind early, soluble fibrillar Aβ species in vitro [19] and effectively reduce the pool of cytotoxic Aβ oligomers by mitigating Aβ-induced neurotoxicity [20]. In a separate study, aggregation kinetics and binding assays demonstrated that the LCO, heptamer formyl thiophene acetic acid (h-FTAA) and its pentameric counterpart, p-FTAA, interacted with distinct pre-fibrillar in vitro Aβ species. Both LCOs exhibited cytoprotective effects against various Aβ assemblies in SH-SY5Y neuroblastoma cells, highlighting their potential to modulate early aggregation pathways [21]. In our previous study, we investigated the binding ability of h-FTAA to recombinant αsyn pre-formed fibrils (PFFs) and used the human embryonic kidney 293 (HEK293) cell model (modified to transiently express native αsyn) to assess the binding of h-FTAA to intracellular αsyn morphotypes induced by PFFs exposure [22]. We also hypothesized that it is probable that h-FTAA could also be detecting early αsyn conformers, in addition to mature morphotypes in vitro.

To validate this hypothesis and to consider the potential of h-FTAA to bind pre-fibrillar protein assemblies, our current study aims to evaluate the fibrillation kinetics of human wildtype-αsyn (huWT-αsyn) and human A53T-mutated αsyn (huA53T-αsyn) using h-FTAA and compare it with the amyloid-specific dye, Thioflavin T (ThT) [23,24]. Furthermore, the HEK293 cell model, genetically modified to stably express native huWT- or huA53T-αsyn, was exposed to either huWT- or huA53T-αsyn PFFs to induce intracellular αsyn aggregation. The induced intracellular αsyn expression and aggregates were validated using specific immunocytochemistry (ICC) labels. In addition, the PFFs exposed HEK293 cells, stably expressing native human αsyn labeled with h-FTAA. The conformational state at the binding sites of the intracellular aggregates was investigated by analyzing the spectral characteristics and fluorescence lifetime profiles.

## 2. Materials and Methods

### 2.1. Preparation and Characterization of huA53T- and huWT-αsyn PFFs

Sample preparation was carried out in the general chemistry laboratory, while the work with fibrillated αsyn was carried out in the Biosafety Level-3 (BSL) prion lab for safety precautions. Thus, 0.5 mg native huWT and huA53T αsyn (rPeptide, Alpha-Synuclein, S-1001; Alpha-Synuclein, A53T Mutant, S-1002) were dissolved and filtered (Thermo Fisher Scientific, Trondheim, Norway, 1 mL plastic syringe, 14-955-456) (GE Healthcare Life Science, Oslo, Norway, Syringe Filter, 6779-0402) (GE Healthcare Life Sciences Whatman, 4 mm Single Use Filter Device, 6779-0402) in 600 μL PBS (Medicago Group, Oslo, Norway, 10-9402-10) in 1.5 mL tubes (Sarstedt, Germany 72.703.600) and gently mixed by pipetting for a homogeneous solution. The protein concentration was quantified with a photometer (NanoPhotometer NP80, IMPLEN (Munich, Germany)), using the extinction coefficient, 5 960 M^−1^·cm^−1^ [25] at 275 nm. For kinetics analysis, native huWT- and huA53Tαsyn were incubated with either h-FTAA or ThT in PBS to a final protein concentration of 50 μM. The synthesis of h-FTAA has been published elsewhere [26]. Fluorophore concentrations were 500 nM for h-FTAA and 10 μM for ThT. Each condition was prepared in a final volume of 50 μL and pipetted in quadruplicate into a 96-well plate (Corning, Norway, 96-Well Half Area Black/Clear Flat Bottom Polystyrene NBS Microplate, 3881). The outer two rows and columns were deliberately not used, as samples in these wells are prone to evaporation. PBS only wells and native protein in PBS were used as controls. The plate was transported into the BSL-3 lab, where another control of already formed αsyn fibrils was added for calibration. One 3 mm glass bead (Sigma-Aldrich/Merck Life Science AS, Oslo, Norway, Solid-glass beads, Z265926) was added to each well containing a sample to promote agitation and fibrillation. MilliQ (MQ) water was added to adjacent wells, as well as in between wells, to protect samples from evaporation. The well plate was sealed with aluminum foil before it was incubated in the Clariostar plate reader (BMG Labtech, Oretenberg, Germany, CLARIOstar Plus) at a temperature of 37 °C and under agitation (5 min of 600 rounds per minute, rpm). The aggregation process was monitored in real-time by collecting fluorescence every 15 min for 40 h. h-FTAA [22,26] and ThT [27] have overlapping spectral excitation and emission profiles, and the same settings were used (excitation 440 nm; emission 470–640 nm). Emissions from “probe only” samples were used as a baseline. Spectral properties of the samples and control PPFs were further analyzed using a Tecan Saphire^2^ fluorescence plate reader (Tecan, Männedorf, Switzerland) and Leica SP8 (Ortomedic, Leica Microsystems, Oslo, Norway).

### 2.2. Data Analysis of the Fibril Aggregation Assay

The raw data in terms of fluorescence spectra from each well was integrated, and the resulting intensity vs. time signal was smoothed using a standard 13-point, third-order Savitzky–Golay moving average filter. The time profiles were baseline corrected with the PBS/dye-only sample data prepared in an identical manner, and after removing evident outliers, the average signal of each sample set was calculated and re-normalized for convenient data fitting and comparison in the associated plots. The time profile of the aggregation kinetics was fitted using the widely used sigmoidal function [27,28]

(1) $$F\left(t\right)=F_{0}+\frac{F_{max}-F_{0}}{1+exp\left[-k_{app}\left(t-t_{\frac{1}{2}}\right)\right]}\;\;\;\;\;\;\;\;\;\;\;\;\;\;\;\;\;,$$

where $F\left(t\right)$ is the collected time series of fluorescence data, and $F_{0}$ and $F_{max}$ are the initial and final plateaus of the sigmoidal curve shape, respectively. The essential parameters for the aggregation kinetics are contained in the following parameters: $k_{app}$ is the apparent growth rate (units of time^−1^), and the time point $t_{1/2}$ defines the inflection point of the sigmoidal shape (50% of $F_{max}-F_{0}$), usually referred to as the half-time value. These can then be used to calculate the so-called lag time, $t_{lag}=t_{1/2}-\frac{2}{k_{app}}$, defining the time point when significant aggregation begins. To reduce the number of free parameters in the fitting, the data points were re-normalized to have $F_{0}=0$. The essential kinetics parameters, along with standard deviation via the quality of the fit, are presented in Table 1 in the results section. The data manipulation, fitting and presentation were carried out using Origin Pro 2025.

### 2.3. Stable Expression of Endogenous huA53T- or huWT-αsyn in HEK293 Cells

The HEK293 cells were grown using Eagle’s Minimum Essential Medium (30-2003, ATCC LGC standard, UK), supplemented with 10% Fetal Bovine Serum (both Sigma Aldrich, Merck Life Science AS, Norway) and maintained in a 37 °C humidified incubator at 5% CO_2_. They were transfected with 4 µg of pcDNA3.1 plasmid containing native huWT- or huA53T-αsyn using lipofectamine 2000 (Invitrogen, Life technologies AS, Oslo, Norway). The expression of the huWT- or huA53T-αsyn gene in the plasmid is driven by the cytomegalovirus promoter. Cells stably expressing the gene were expanded and grown based on the selection marker, Neomcyin, conferred to them through the plasmid. 400 µg/mL Geneticin (G418) antibiotic (Gibco, Thermofisher Scientific, Oslo, Norway) was used for selecting the stable cells post transfection.

### 2.4. Exposure of Stable HEK293 Cells to huWT- or huA53T-αsyn PFFs

Both huWT- and huA53T-HEK293, together with the control HEK293 cells, were plated in a 12-well plate (Corning, VWR, International AS, Oslo, Norway). Before PFFs transfection, the huαsyn PFFs were briefly sonicated for 5 min using Bandelin Sonorex at 35 kHz. For a reaction mixture of 100 μL per tube, 0.5 μL of Lipofectamine 2000 was diluted in 50 μL of OptiMEM (Gibco, Thermofisher Scientific, Oslo, Norway) and incubated for 5 min at room temperature. Simultaneously, PFFs were diluted in 50 μL of OptiMEM and incubated for 5 min at room temperature. The lipofectamine2000-OptiMEM mix was added to the PFFs-OptiMEM mix, incubated for 15 min at room temperature, and added to the cells at a final PFFs concentration of 1 μM. Post 48 h of incubation, the cells were washed with PBS (Gibco, Thermofisher Scientific, Oslo, Norway) to remove any extracellular fibrils, treated with 0.25% Trypsin-EDTA (Sigma Aldrich, Merck Life Science AS, Norway)), subcultured into μ-slide ibidi plates (IB80806, IB81816, Inter instrument AS, Høvik, Norway), and were further incubated for a week (37 °C, 5% CO_2_). Immunocytochemistry was performed at two different time points, day 6 and day 8 post initial PFFs exposure, and the fluorescence images were acquired on day 7 and day 9. (It was found in separate control measurements that 5–6 days was necessary to observe aggregation.)

### 2.5. Immunocytochemistry

The cells were rinsed with PBS and fixed with 4% paraformaldehyde (PFA: P60148, for 20 min at room temperature. Following washes with PBS (2×), the cells were treated with the blocking buffer (3% BSA in PBS, 0.1% Triton X-100) and incubated for 1 h at room temperature (all Sigma Aldrich, Merck Life Science AS, Norway). The cells were either treated with anti-αsyn monoclonal antibody (1:500, 32-8100 Syn 211, Invitrogen, Life technologies AS, Oslo, Norway) alone or counterstained with a mix of Syn211 (1:500) + anti-αsyn pS129 antibody (1:2000, EP1536Y Abcam) and incubated overnight (4 °C). Following washes with PBS (3 × 10 min), the cells were incubated with secondary antibody for 1 h at room temperature. The secondary antibodies used included goat anti-mouse conjugated to Alexa Fluor 488 (1:2000, A-21121 Invitrogen, Life Technologies AS, Oslo, Norway) or a mix of Alexa Fluor 488 (1:2000) + goat anti-rabbit conjugated to Alexa Fluor 647 (Alexa Fluor 647, 1:2000, A-21245 Invitrogen, Life Technologies AS, Oslo, Norway). Post incubation, the cells were washed with PBS (3 × 10 min). Based on the experimental setups, cells were stained with 200 nM Mitotracker orange CMTMRos, (M7510 Invitrogen, Life Technologies AS, Oslo, Norway) (before fixation with PFA) and incubated for 45 min at room temperature. Cell nuclei were stained using 4′,6-diamidino-2-phenylindole (1 μg/mL DAPI, 62248 Invitrogen, Life Technologies AS, Oslo, Norway), incubated for 5 min. After rinsing with PBS (2×), the cells were imaged using Zeiss 800 Alryscan Confocal Microscope (Carl Zeiss AS, Oslo, Norway). Images were analyzed using Image J version 1.53t software.

### 2.6. Immunoblotting

Immunoblotting was performed to assess endogenous expression of native huαsyn in HEK293 cells, according to the protocol cited in [22].

### 2.7. Staining with h-FTAA, pS129-αsyn and Spectral Imaging, FLIM of h-FTAA Bound Intracellular Aggregates

Cells were washed with PBS and then fixed with 4% PFA for 20 min at room temperature. Following washes with PBS (2×), 1 μM h-FTAA was added to the cells and incubated for 1 h at room temperature. The cells were washed with PBS (2×) and then imaged using Leica SP8 (Ortomedic, Leica Microsystems, Oslo, Norway) with single-molecule detection and multiphoton laser confocal microscope. h-FTAA was excited at 475 nm, and the spectral emission scans were acquired by setting the emission scan range from 490 to 695 nm, divided into 35 detection steps, resulting in a λ-detection step size of 6 nm. The Alexa Fluor 647 (pS129-αsyn) was excited at 650 nm, and the emission scans were measured by setting an emission scan range from 658 to 745 nm, divided into 28 detection steps, spaced 3 nm apart. For measuring FLIM and analyzing fluorescence lifetime plots of h-FTAA bound aggregates, PicoQuant’s Sym Pho Time 64-bit version software was used with a pulsed laser set at 40 Mhz and the laser intensity maintained between 2 and 5%.

### 2.8. Confocal Microscopy, FLIM and TEM of hu-αsyn PFFs

huWT- or huA53T-αsyn PFFs (50 µM) were mixed with h-FTAA (500 nM) and microscope slides were prepared with 2 µL of the respective stained samples. The stained samples were imaged using Leica SP8 with single-molecule detection and multiphoton laser confocal microscope by exciting h-FTAA at 470 nm, setting the emission scan range from 490 to 650 nm. For FLIM acquisition and lifetime distribution analysis, PicoQuant’s SymphoTime 64-bit version was used. Lifetime distributions within images were fitted to a standard Gaussian function $A\cdot e^{\left(-{\left(\tau_{i}-\mu \right)}^{2}/2\sigma^{2}\right)}$, where *μ* is the average lifetime and *σ* the standard deviation of the associated Gaussian normal distribution.

Negative stain transmission electron microscopy samples were prepared by aliquoting 5 μL of αsyn fibril samples (at the end of the fibril formation reaction) onto 400-mesh carbon-coated copper grids (Carbon-B, Ted Pella Inc., Redding, CA, USA). The samples were incubated for 2 min for fibrils to adsorb to the grid surface. Excessive solutions were blotted off with filter paper. The residual buffer was rinsed off by one wash of 5 μL of milliQ water. The grids were stained with 2% uranyl acetate for 30 s before the grid was blotted dry and was left to dry overnight. Transmission electron microscopy (TEM) imaging was performed using a Jeol JEM1400 Flash TEM microscope (JEOL Nordic AB, Sollentuna, Sweden) operating at 80 kV.

## 3. Results and Discussion

### 3.1. Endogenous Stable Expression of Monomeric hu-αsyn in HEK293 Cells

**Figure 1:**
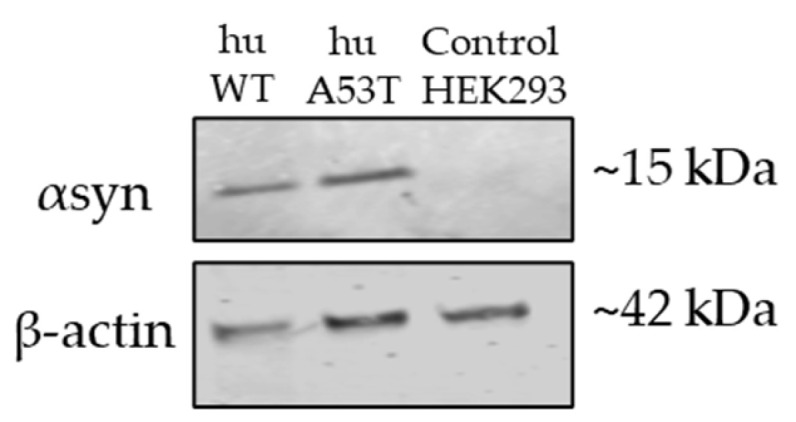
Representative Western blot showing endogenous αsyn protein expression in HEK293 cells, stably expressing native huWT- or huA53T-αsyn.

In order to have continuous native αsyn protein expression in cells that could potentially act as a template for further protein aggregation [29], the HEK293 cells were transfected with human-WT-αsyn (huWT-HEK293) or human-A53T-αsyn (huA53T-HEK293). The transfected cells were cultured in EMEM growth medium containing Geneticin to ensure the preferential survival and selection of those cells that had successfully incorporated the hu-αsyn gene for stable expression. The hu-αsyn protein expression in the huWT- and huA53T-HEK293 cells was assessed by Western blotting using an anti-αsyn monoclonal antibody (Syn211). Expectedly, the protein bands as shown in Figure 1 were detected in huWT- and huA53T-HEK293 cells at ~15 kDa, whereas no protein band was seen in non-transfected control HEK293 cells (control HEK293).

**Figure 2:**
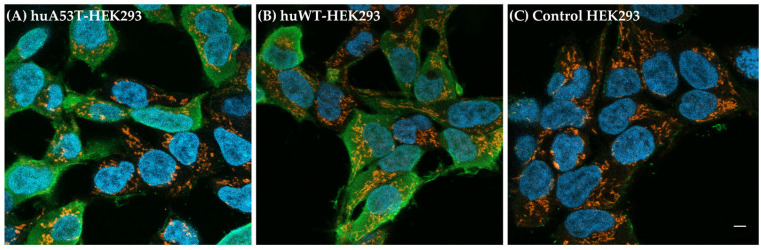
Representative fluorescence images showing endogenous hu-αsyn protein expression in HEK293 cells, stained with anti-αsyn antibody (Syn211). (**A**) huA53T-HEK293 cells and (**B**) huWT-HEK293 cells showing intracellular αsyn localization in cytosol (green). (**C**) Control HEK293 cells present minimal background when stained with Syn211. Costaining: DAPI (blue). Mitotracker Orange (orange). The scale bar represents 10 μm.

As an additional control measure, immunocytochemistry was performed to assess the intracellular localization of monomeric hu-αsyn in the transfected cells. The huWT-, huA53T-HEK293 cells, including the control HEK293 cells, were stained with Syn 211 antibody, and the corresponding fluorescence signal from intracellular hu-αsyn was detected using Alexa Fluor 488 conjugated to a secondary antibody. Representative confocal laser scanning microscope images are depicted in Figure 2A,B. The huA53T- and huWT-HEK293 cells exhibited strong fluorescence from Alexa Fluor 488 (green), indicating the presence of hu-αsyn in both cell types. The non-transfected control HEK293 cells showed a few weak background spots that were probably from some unspecific Alexa Fluor 488 localization and thus, no evident αsyn expression (Figure 2C). The cells were in this experiment also probed with DAPI (blue), which stains the nucleus, and Mitotracker orange CMTMRos (orange) for labeling mitochondria to qualitatively assess intracellular localization of endogenous αsyn in cells. As shown in Figure 2A,B, the native αsyn localizes mostly to the cytosol. These observations taken together confirm the expression of hu-αsyn in huWT- and huA53T-HEK293 cells.

### 3.2. Spectral Fibrillation Kinetics of huWT- and huA53T-αsyn PFFs with hFTAA and ThT

**Figure 3:**
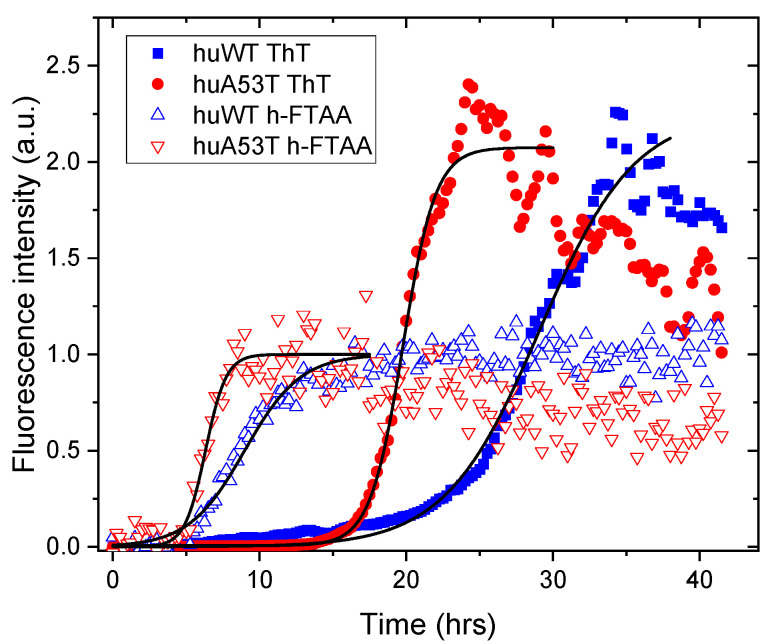
Real-time fibrillation kinetics of huWT- (blue) and huA53T-αsyn (red) PFFs (50 μM) monitored using amyloid-specific fluorescent probes. Open symbols are h-FTAA (500 nM), and filled symbols are ThT (10 μM). The spectral emission was measured at time points every 15th min, and the integrated signal is presented. The samples were excited at l_ex_ = 450 nm, and emissions were collected in the range l_em_ = 470–620 nm, selected to suit both the ThT and h-FTAA spectra. Black solid lines are fits described in the text and Section 2.2.

To prepare for cell seeding experiments, a spectroscopic assay was performed to investigate the fibrillation kinetics of αsyn in vitro fibrils using the amyloid-specific fluorescent ligands ThT and h-FTAA. The former is the standard for detecting mature amyloid fibrils, such as, for example, aggregated αsyn variants [23,24] whereas h-FTAA has also been suggested to be sensitive to early aggregates such as Aβ oligomers [21]. Thus, huWT and huA53T-αsyn PFFs were produced by incubating monomeric protein under agitation at 37 °C for 40 h as described in detail in Section 2.1. The fibrillation kinetics curves presented in Figure 3 are typical for ThT (filled symbols). Notably, the aggregation of mature fibrils has an earlier onset for the A53T mutation as expected [23,24]. It should be pointed out that such time-lapses are sensitive to temperature, protein concentration, and salt [25], as well as the amount of ThT, although in the latter case for considerably higher ThT concentrations than used herein [30].

Interestingly, the data of the h-FTAA assays (Figure 3, open symbols) demonstrate that the pertinent fluorescence increase here is considerably earlier than for ThT, indicating sensitivity of early pre-fibrillar aggregates. Moreover, also in this case, the onset of the fluorescence increase for the huA53T mutant is distinctly earlier than for huWT-αsyn, mimicking the ThT results. The solid black lines in Figure 3 represent fits to the aggregation time traces with the widely used two-parameter sigmoidal function, where the essential parameters $k_{app}$ (apparent growth rate) and $t_{1/2}$ (half-time) are found from the curve shape, and $t_{lag}$ (lag-time) is deduced from those two parameters, as summarized in Table 1 (see Section 2.2 for details).

**Table 1:** Parameters of the aggregation kinetics deduced from fitting of the time-lapses in Figure 3 ^1^.

Sample	$k_{app}$ (hr^−1^)	$t_{1/2}$ (hr)	$t_{lag}$ (hr)
huWT ThT	0.310 ± 0.008	29.1 ± 0.1	22.6 ± 0.6
huA53T ThT	0.898 ± 0.004	19.8 ± 0.1	17.6 ± 0.1
huWT h-FTAA	0.53 ± 0.03	9.0 ± 0.1	5.2 ± 0.3
huA53T h-FTAA	1.4 ± 0.2	6.3 ± 0.1	4.9 ± 0.7

^1^ The errors are relatively small due to the fit of the function to an average of experimental time traces.

One can conclude that the time traces of all samples give good fits to the standard sigmoidal model (Section 2.2) except for the case of huWT with ThT, which has the longest lag phase as well as the slowest growth rate. Here an initial process can be discerned as a weak shoulder around 10–15 hrs after onset of the reaction from a visual inspection of the pertinent plot in Figure 3. This may imply secondary aggregation processes that require considerably more experiments using various concentrations and procedures to elucidate [31]. This is beyond the scope of the present study that focuses on their impact as seeds for the cell models. It is also noteworthy that the growth rate of huWT is approximately 1/3 of the rate for huA53T for both h-FTAA and ThT, indicating that the A53T fibrils have more efficient elongation and nucleation rates in accordance with previous reports [24]. This also suggests that both ligands, h-FTAA and ThT, monitor the same processes; however, ThT is sensitive to the mature fibrils, whereas h-FTAA also responds to earlier pre-fibrillar forms, as has also been observed for Aβ [26]. Further details on the h-FTAA emission spectra of early and mature aggregates along with the FLIM results of in vitro PFFs will be presented and discussed below.

### 3.3. Confocal Microscopy, TEM Images, Emission Spectra, FLIM of huWT-and huA53T-αsyn PFFs

**Figure 4:**
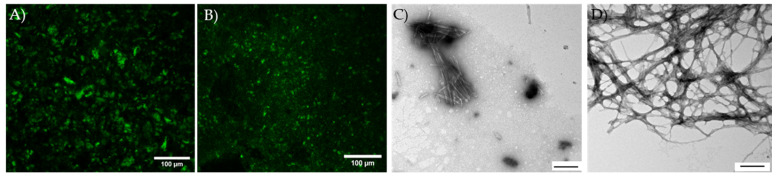
Confocal microscopy images illustrating aggregate formation in (**A**) huWT- and (**B**) huA53T-αsyn fibrils (50 μM) stained with h-FTAA (500 nM). Images are taken with the Leica SP8 with a 20×/dry objective. l_ex_ = 470 nm; l_em_ = 490–650 nm. Transmission electron microscopy images of (**C**) huWT and (**D**) huA53T-αsyn fibrils (50 μM) negatively stained with uranyl acetate. The scale bar is 100 µm in (**A**,**B**) and 200 nm in (**C**,**D**).

To compare PFFs with fibril-ligand complexes detected in the αsyn cell models, confocal hyperspectral fluorescence microscopy in combination with fluorescence lifetime imaging (FLIM) was performed on the huWT- and huA53T-αsyn fibrils stained with h-FTAA. The morphology of huWT- and huA53T-αsyn fibrils with 500 nM of h-FTAA, as harvested at the end of the kinetic measurement (Figure 3) with 100× excess of protein, is shown in Figure 4A,B. It can be noted that the huWT variant shows larger clustered aggregates of fibrils than for huA53T fibrils. The intensity of the latter is somewhat bleaker. Formation of amyloid fibrils by huWT and huA53T-αsyn was also confirmed using transmission electron microscopy (TEM), where both variants showed significant clustering of formed filaments (Figure 4C,D).

**Figure 5:**
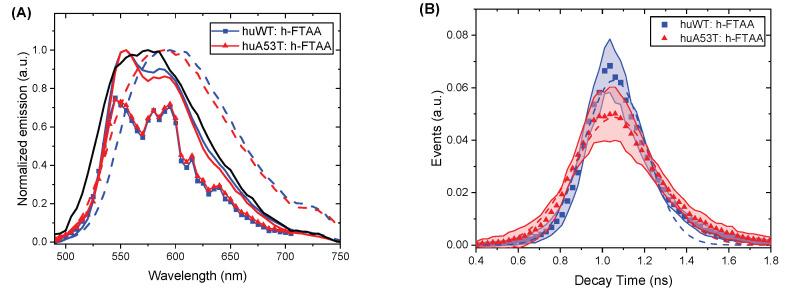
(**A**) Emission spectra of αsyn fibrils stained with h-FTAA and measured as described in the text. Color code: blue (huWT); red (huA53T); black (h-FTAA only). l_ex_ = 470 nm. The emission amplitudes have been normalized to conveniently compare the spectral profiles. (**B**) Fluorescence lifetime distributions from huWT (blue) and huA53T (red)-αsyn fibrils (50 μM), stained with h-FTAA (500 nM) as described in the text. The shaded area indicates the standard deviation for 20 ROIs, and dashed curves indicate fits as described in the text. Settings: l_ex_ = 475 nm; l_em_ = 510–620 nm.

Using the spectral scanning feature of the fluorescence microscope, the pertinent emission spectra of Figure 4A,B were obtained, shown in Figure 5A (symbols) from the average of 20 ROIs of images. The blue and red color codes represent huWT and huA53T fibrils, respectively, and as can be seen, both PFFs give very similar spectral profiles.

Added to the plot in Figure 5A are also spectral profiles using a Tecan plate reader at different loadings of h-FTAA relative to 1 mM of fibrils of the two cases. The solid lines represent 2.5× fibrils in excess of h-FTAA, whereas the dashed lines represent 2.5× excess of h-FTAA relative to PFFs. The black solid line is for h-FTAA only (1 mM). Thus, at higher h-FTAA loading, the spectral profile is clearly red-shifted by 20–25 nm and does not resolve the vibrational peaks at approximately 550 and 580 nm observed both in the microscope spectral data and in the plate reader at low h-FTAA loadings. Conclusively, at low relative loadings of h-FTAA, the strongest binding site is populated, giving a resolved double-peak emission. With excess of h-FTAA, secondary binding sites are also populated, giving a red-shifted and more featureless spectrum, while the double peak is diminished. It can be anticipated that in a system in a progressing fibrillation process, spectral components with and within these extremes will be superimposed on a broader, more featureless spectrum.

Lifetime distributions, shown in Figure 5B, showed similar fluorescence lifetime profiles for both PFF genotypes, with average lifetimes around 1 ns. A standard Gaussian distribution function was used to fit the data, revealing a mean lifetime of 1.06 ns for both PFFs. It also revealed a greater spread for the huA53T PFF variant, with the (variance) width parameter σ (Figure 5B) being 0.20 ns, compared to 0.15 ns for huWT (FLIM results are summarized in Table 2).

### 3.4. huA53T- or huWT-αsyn Protein Aggregation in Stable HEK293 Cells

For assessing intracellular protein aggregation in HEK293 cells that have been modified to stably express monomeric hu-αsyn, they were exposed to 1 μM of huA53T- or huWT-αsyn PFFs for 48 h. After 48 h of fibril exposure, the cells were subcultivated and further incubated for a week. The ensuing intracellular protein aggregation was assessed through conventional immunocytochemical (ICC) staining performed at two different time points—day 6 and day 8 post initial PFFs exposure—and the fluorescence images were taken on day 7 and day 9, respectively. The cells were counterstained with Syn211 and anti-αsyn pS129 (pS129-αsyn) antibodies for detecting native αsyn as well as induced αsyn aggregates in cells. One of the key pathological features of αsyn aggregation is post-translational modification (PTM) with phosphorylation of Ser129 (pS129) as the main PTM [32]. This pathological marker is widely used as a control to assess αsyn aggregation in brain tissue sections as well as in cell models of PD [33,34]. To this end, anti-αsyn pS129 antibody was used as a control to visualize αsyn aggregation in the HEK293 cells, modified to stably express hu-αsyn using a confocal laser scanning microscope. Syn211 + pS129-αsyn were detected by probing the cells with secondary antibodies conjugated to Alexa Fluor 488 (green) and Alexa Fluor 647 (red).

**Figure 6:**
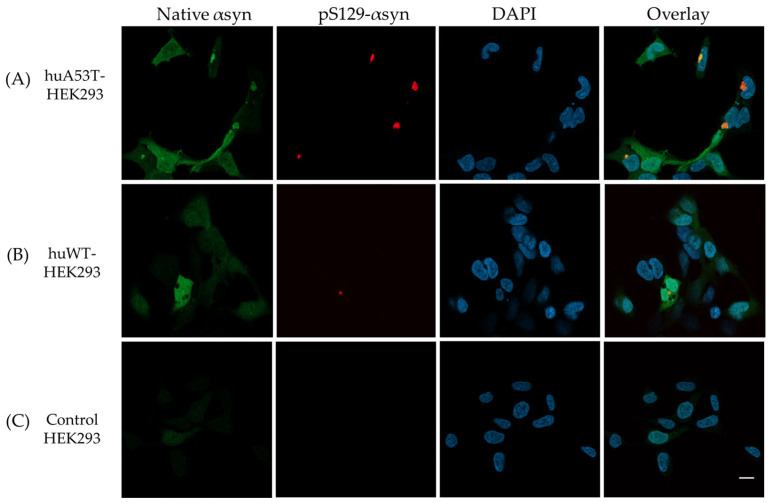
Representative fluorescence images of (**A**) huA53T-HEK293 and (**B**) huWT-HEK293 cells, exposed to 1 μM huWT-αsyn PFFs and labeled with Syn211 (green, 1:500) + pS129-αsyn (red, 1:2000). (**C**) Control HEK293 cells, also exposed to huWT-αsyn PFFs, exhibit minimal background from Syn211 + pS129-αsyn. Images were acquired on day 7 following initial PFFs exposure, at 40× magnification. Cell nuclei were stained with 1 μg/mL DAPI (blue). The scale bar represents 10 μm.

As shown in Figure 6A,B, the representative fluorescence images of huWT- or huA53T-HEK293 cells, imaged on day 7, exhibit intracellular αsyn aggregation (red) when transfected with huWT-αsyn PFFs. Moreover, huA53T-HEK293 cells present more pronounced aggregation (Figure 6A) compared to huWT-HEK293 cells (Figure 6B) when visually inspected. Additionally, nuclear staining with DAPI (blue) provided an insight into potential localization of the aggregates within the cells. As apparent from Section 3.1, the huWT- and huA53T-HEK293 cells here also showed intracellular expression of native hu-αsyn (green), which was localized to the cytosol, as opposed to the induced intracellular αsyn aggregates, which appeared to form clusters near the nucleus, consistent with earlier studies [35,36,37]. As expected, no fluorescence signal was detected for pS129-αsyn in the control HEK293 when imaged at the same time point as the huWT- and huA53T-HEK293 cells post huWT-αsyn PFFs exposure (Figure 6C). The images taken on day 9, presented in Figure S2A, show similar intracellular aggregation trends in huA53T-HEK293 as opposed to the huWT-HEK293 cells (Figure S2B), while no obvious protein aggregation was detected in the control HEK293 cells (Figure S2C).

It is noted that the native αsyn (stained with Syn211) shows more homogenous localization across the cytoplasm with pS129-positive puncta appearing as clusters within the Syn211-stained regions (Figure 6). This observation is consistent with the hypothesis that externally applied PFFs most likely act as seeds that recruit monomeric αsyn to induce aggregation in cells [38,39]. The huA53T-HEK293 cells exhibit enhanced aggregation in comparison to huWT-HEK293 cells, which display few pS129-positive puncta. The enhanced aggregation observed in huA53T-HEK293 cells is possibly linked to the transfected gene encoding the A53T-mutated form of native αsyn. The A53T mutation, which is one of the dominant mutations linked to familial forms of PD, is well known to display an enhanced aggregation phenotype [24,40,41,42].

**Figure 7:**
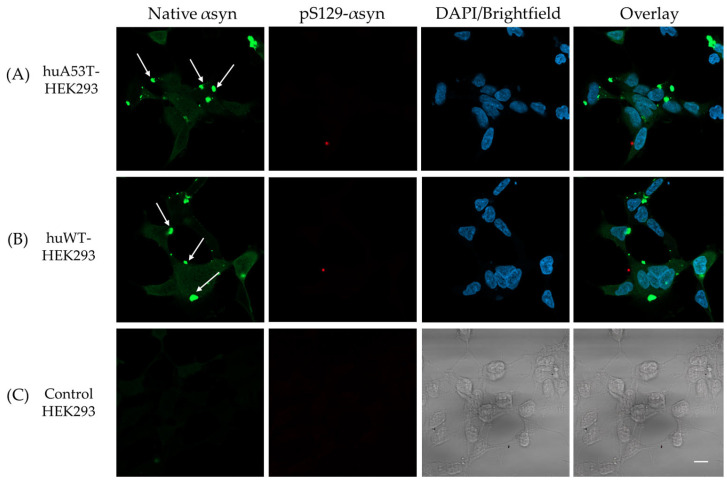
Representative fluorescence and brightfield images of (**A**) huA53T-HEK293, (**B**) huWT-HEK293 cells and (**C**) control HEK293 cells, exposed to 1 μM huA53T-αsyn PFFs, counterstained with Syn211 (green, 1:500) + pS129-αsyn (red, 1:2000). (1 μg/mL) DAPI (blue) was added for labeling of cell nuclei in (**A**,**B**). Images were acquired on day 7 post initial fibril seeding. The scale bar represents 10 μm, 40× objective used.

Interestingly, the huWT-, huA53T-HEK293 cells exposed to huA53T-αsyn PFFs, when counterstained with the Syn211 + pS129-αsyn antibodies and imaged on day 7 post PFFs transfection, showed aggregation, but not prominently pS129-positive puncta (Figure 7A,B) as opposed to previous observations with huWT-αsyn PFFs (Figure 6A,B). Notably, the Syn211 staining presented distinct deposits, indicated with white arrows in Figure 7A,B. The initial PFF seeds exogenously added to the cells are not likely visible here, since the cells exposed to fibrils were subcultured to get rid of extracellular fibrils. It could be likely that the huA53T-αsyn PFFs might have rendered the huWT- and huA53T-HEK293 cells to possibly exhibit some early aggregation patterns. This observation is distinct from the ones shown in Figure 2A,B wherein the Syn211 staining in the huWT- and huA53T-HEK293 cells is mostly homogenous with no apparent deposits seen. One might also speculate that the huA53T-αsyn fibrils exposed cells, modified to stably express hu-asyn, exhibit early aggregation patterns with possibly only a few evolved αsyn morphotypes. This assumption is based on comparative fibrillation kinetics, which indicated that huA53T-αsyn undergoes fibrillation earlier than huWT-αsyn, as detected by ThT. Further supporting literature indicates that the A53T mutation linked to early-onset familial PD facilitates the formation of oligomeric, protofibrillar species, exhibiting increased phenotypic severity [43,44,45,46].

As illustrated in Figure 7C, the control HEK293 cells did not exhibit any aggregation events. The confocal images in Figure S3A,B, taken on day 9 post initial huA53T-αsyn PFFs exposure, showed similar aggregation events in huWT- and huA53T-HEK293 cells, while no apparent fluorescent signal was detected for either Syn211 or pS129-αsyn in control HEK293 cells (Figure S3C). Conclusively, these observations suggest that the huA53T-αsyn PFFs (Figure 7A,B) could possibly be inducing early aggregation/fibrillation as opposed to huWT-αsyn PFFs (Figure 6A,B) in HEK293 cells genetically engineered to stably express native hu-αsyn.

### 3.5. Spectral Features and FLIM of h-FTAA, Bound to Intracellular hu-αsyn Aggregates, Induced by huWT- or huA53T-αsyn PFFs

#### 3.5.1. h-FTAA-Labeling of Intracellular Aggregates, Induced by αsyn PFFs

h-FTAA is a well-established, versatile probe widely used for detecting various amyloid polymorphs in transgenic mouse models [47,48] and in pathological brain tissue sections [18]. It has also been reported that h-FTAA is able to bind to prefibrillar Aβ species [21]. In order to assess whether the intrinsic chemical environment surrounding intracellular aggregate morphology influences the formation of potential earlier aggregates, the HEK293 cells, stably expressing huWT- or huA53T-αsyn, were exposed to 1μM huWT- or huA53T-αsyn PFFs. The ensuing intracellular aggregates were stained with 1 μM h-FTAA and counterstained with pS129-αsyn antibody. The representative fluorescence images taken on day 7 post initial fibril seeding are presented in Figures S4 and S5. The h-FTAA staining in huWT- and huA53T-HEK293 cells (Figures S4 and S5A,B) shows enhanced fluorescence with minimal background, making h-FTAA a sensitive ligand for characterizing polymorphic protein aggregates as evidenced by literature [48]. Counterstaining with pS129-αsyn (red) served as a control to visualize phosphorylated intracellular aggregate formation. When visually compared to pS129-αsyn staining, the h-FTAA exhibits more pronounced staining, suggesting that h-FTAA could be plausibly detecting initial fibrillar aggregates as opposed to pS129-αsyn, which typically binds to matured aggregates. In contrast, the control HEK293 cells, which were also seeded with αsyn PFFs, showed minimal background when probed with h-FTAA (Figures S4 and S5C). The fluorescence images taken on day 9 (Figures S6 and S7A) showed some overlapping stained regions of h-FTAA and pS129-αsyn in huA53T-HEK293 cells, whereas weaker fluorescent signals of h-FTAA + pS129-αsyn were detected from huWT-HEK293 cells (Figures S6 and S7B). As expected, no visible fluorescent signals from either pS129-αsyn or h-FTAA were detected in control HEK293 cells (Figures S6 and S7C).

#### 3.5.2. Emission Profiles and FLIM of h-FTAA Bound Intracellular Aggregates, Induced by huWT- or huA53T-αsyn PFFs

**Figure 8:**
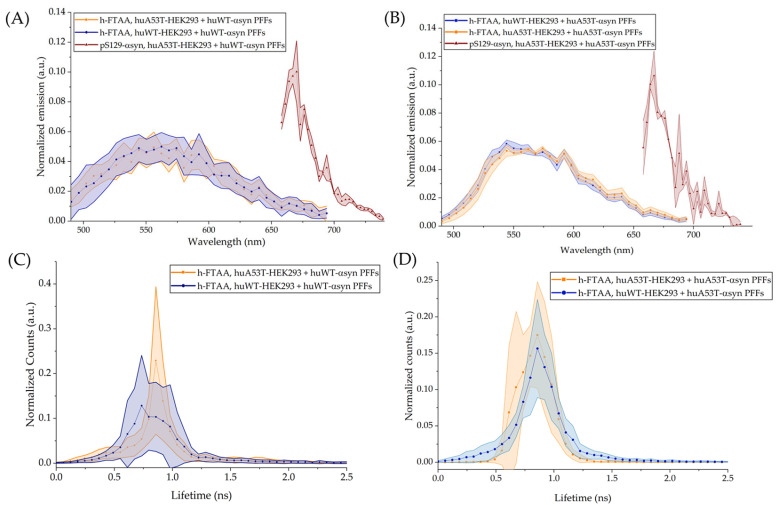
Spectral emission profiles (**A**,**B**) and fluorescence lifetime distributions (**C**,**D**) of h-FTAA, bound to aggregates in huWT- and huA53T-HEK293 cells, when exposed to 1 μM huWT-(**A**,**C**) or huA53T- (**B**,**D**) αsyn PFFs. These plots correspond to the fluorescence and FLIM images taken on day 7, following initial exposure to fibrils. The λ_ex_ for h-FTAA was at 475 nm, and λ_em_ was measured using the 490–695 nm range. The λ_ex_ for Alexa Fluor 647 (pS129-αsyn probing) was at 650 nm, and λ_em_ was recorded using the 658–745 nm range. The shaded regions in the emission and in the fluorescence lifetime plots represent the standard deviation from seven ROIs.

Spectral emission plots were generated by selecting various regions of interest (ROIs) corresponding to h-FTAA-stained intracellular aggregates, including those positive for pS129-αsyn. Data were acquired from three distinct imaging regions within each well of the µ-slide well plates. Seven ROIs were selected for analysis, and the emission plots were background corrected. The corresponding emission profiles are presented in Figure 8A,B. The shaded regions in the emission plots (Figure 8A,B) represent the standard deviation from seven ROIs and were normalized to the total area of the spectrum. Similar emission profiles were observed for both cell types, with characteristic maxima emerging around 545–585 nm with a red-shifted trend as reported previously [22]. To assess whether there is a spectral emission overlap between h-FTAA and pS129-αsyn, the fluorescence emission was recorded for Alexa Fluor 647, which binds to the pS129-αsyn antibody. The samples were excited at 650 nm, and the corresponding emission plot in Figure 8A,B (brown shaded emission plots) shows the emission maximum for pS129-αsyn around 670 nm, well separated from the h-FTAA emission. The emission profiles of h-FTAA binding to intracellular aggregates, generated on day 9 post initial fibril exposure, also showed a similar trend for both cell types (Figure S8A,B).

When compared to the spectral profiles of h-FTAA binding to fibrils using the Tecan plate reader (Figure 5A), the emission peak observed for the intracellular aggregates seemed to align more towards a red-shifted trend, possibly suggesting that h-FTAA could be binding to multiple conformers of αsyn through more than one binding site. Notably, the spectral features measured in the cellular milieu were broadened with fewer features compared with the pristine PFFs. This is likely, as fibrillation within the cellular system is probably more heterogeneous with a multitude of αsyn pre-fibrillar and fibrillar structures. Moreover, cellular compartments also offer a variety of local surroundings with different impacts on fluorescence lifetime. Hence, a spectral superposition of a multitude of spectra will be the result, and because of the red-to-blue shift upon more mature aggregates (Figure 5A), a spectral broadening is expected.

FLIM is a highly sensitive imaging technique that leverages the fluorescence decay of fluorophores to sensitively detect the conformational changes driven by their intrinsic molecular environment [49]. FLIM has been employed in assessing protein aggregation states in in vitro models [50,51]. For assessing the molecular environment around the h-FTAA-aggregate complex, the FLIM images were recorded on day 7 for the h-FTAA-bound intracellular aggregates by exciting the samples at 475 nm. Fluorescence lifetime distributions of h-FTAA–bound intracellular aggregates, seeded with huWT-αsyn PFFs (Figure 8C) or huA53T-αsyn PFFs (Figure 8D), were derived from representative FLIM images (Figures S9 and S10A,B) acquired on day 7, using the same stained regions previously selected for emission spectral analysis. The shaded regions in Figure 8C,D represent the standard deviation from seven ROIs selected from the stained regions. Fluorescence lifetime distributions were fitted to a Gaussian function, and the ensuing mean fluorescence lifetime (μ) with their associated peak widths (2σ) are summarized in Table 2.

**Table 2:** Mean fluorescence lifetime values (μ) and their associated peak widths (2σ) of h-FTAA binding to intracellular aggregates, induced by huWT- or huA53T-αsyn PFFs.

PFFs + Cell Type, h-FTAA Staining in PFFs Seeded Cells	Mean Fluorescence Lifetime (μ), ns	Peak Width (2σ), ns
Day 7 imaging:		
huWT-αsyn PFFs + huWT-HEK293	0.81 ± 0.005	0.37 ± 0.01
huWT-αsyn PFFs + huA53T-HEK293	0.87 ± 0.004	0.14 ± 0.008
huA53T-αsyn PFFs + huWT-HEK293	0.87 ± 0.004	0.29 ± 0.008
huA53T-αsyn PFFs + huA53T-HEK293	0.83 ± 0.003	0.3 ± 0.006
Day 9 imaging:		
huWT-αsyn PFFs + huWT-HEK293	0.68 ± 0.004	0.3 ± 0.009
huWT-αsyn PFFs + huA53T-HEK293	0.76 ± 0.003	0.29 ± 0.006
huWT-αsyn PFFs + huA53T-HEK293, (h-FTAA labeled, pS129 positive puncta)	0.71 ± 0.003	0.26 ± 0.005
huA53T-αsyn PFFs + huWT-HEK293	0.75 ± 0.003	0.34 ± 0.008
huA53T-αsyn PFFs + huA53T-HEK293	0.75 ± 0.003	0.30 ± 0.006

Following huWT-αsyn PFFs seeding, the mean lifetime (µ) was found to be 0.87 ± 0.004 ns for h-FTAA-labeled aggregates in huA53T-HEK293 cells. In huWT-HEK293 cells seeded with huWT-αsyn PFFs, the Gaussian-fitted fluorescence lifetime distribution for h-FTAA–stained aggregates gave a broader distribution with the mean centered around 0.81 ± 0.005 ns. This observation lies in line with our previous findings, reported in [22]. A broader lifetime distribution observed for h-FTAA in huWT-HEK293 cells seeded with huWT-αsyn fibrils plausibly hints at the presence of heterogeneity in the molecular environment surrounding the ligand, similar to the spectral features discussed above. Since the fluorescence lifetime of a fluorophore is sensitive to its external environment, the additional contribution from the cellular milieu, in terms of pH, polarity or subcellular compartments, might also be influencing the variability observed in the lifetime distributions [52]. The fluorescence lifetime plots generated from the representative FLIM images (Figure S9C,D) captured on day 9 are shown in Figure S8C. Notably, the µ for h-FTAA probed aggregates in huWT-HEK293 cells seeded with huWT-αsyn fibrils was computed to be 0.68 ± 0.004 ns. The µ of h-FTAA binding to aggregates in the same cell type is reduced relative to the values recorded on day 7. While, in huA53T-HEK293 exposed to huWT-αsyn PFFs, the µ was 0.76 ± 0.003 ns. Particularly, the mean lifetime for h-FTAA-stained, pS129-αsyn-positive aggregates was 0.71 ± 0.003 ns. In huWT-HEK293 cells seeded with huA53T-αsyn PFFs, the fluorescence lifetime distribution of h-FTAA binding to aggregates, when fit to a Gaussian function, gave a µ of 0.87 ± 0.004 ns. The mean fluorescence lifetime of h-FTAA-labeled aggregates (including the h-FTAA-labeled, pS129-αsyn positive puncta) was found to be 0.83 ± 0.003 ns in huA53T-HEK293 cells when exposed to huA53T-αsyn. FLIM images (Figure S10C,D) were also recorded on day 9 following huA53T-αsyn fibril exposure. Notably, the associated fluorescence lifetime distributions of h-FTAA-probed aggregates in huWT- and huA53T-HEK293 cells (Figure S8D), when fit to a Gaussian function, had a µ of 0.75 ± 0.003 ns. The µ for the h-FTAA observed here appears to be reduced from the µ recorded for the same cell type on day 7.

These varying parameters appear to be significantly different from the values recorded on day 7 for the same cell type and seeding. The difference in mean lifetime values over time observed here could likely be hypothesized based on two possibilities: first, it is probable that the continuous endogenous αsyn expression in cells persistently gets recruited by PFFs. Second, maybe depending upon which type of PFF (the huWT- or huA53T-αsyn in this case) induces aggregation, the progressive nucleation of protein over time leads to the formation of a heterogenous population of aggregates, which is possibly reflected in the difference in lifetime seen over time [3,53].

Several studies have reported temporal shifts in the fluorescence lifetime of ligands bound to protein aggregates, reflecting changes in aggregate structure [54,55]. Aligning with the literature, the h-FTAA-labeled, pS129-co-labeled αsyn inclusions display different lifetime distributions in brain tissue sections of PD and MSA patients [18]. The fluorescence lifetime distribution of h-FTAA bound to intracellular aggregates in huWT- and huA53T-HEK293 cells, induced by either huWT- or huA53T-αsyn PFFs, exhibited variable mean lifetimes that appeared shorter than those observed for h-FTAA binding to the corresponding huWT- or huA53T-αsyn PFFs (Figure 5B), consistent with our previous findings [22]. These observations collectively suggest the possible changing molecular microenvironments around the h-FTAA-aggregate complex as opposed to h-FTAA-bound PFFs, reflecting a trivial conformational-based shift in the mean lifetime of the h-FTAA-bound aggregates. Taken together, these results may suggest that the intrinsic cellular milieu could be plausibly contributing to the fibrillation process/formation of polymorphic aggregates. This is supported by subtle changes in fluorescence lifetime for h-FTAA-bound intracellular aggregates when measured across time points. It should be pointed out that it seems difficult to spectroscopically discriminate between aggregate types using the h-FTAA ligand in living cell models, but it can be a useful indicator of early aggregation.

## 4. Conclusions

This study highlights the utility of h-FTAA as a conformation-sensitive probe for tracking αsyn aggregation, particularly since the early, oligomeric stages can be relevant for disease progression. By comparing huWT and huA53T-αsyn, we confirmed that the A53T mutation accelerates fibrillation kinetics, consistent with its association with familial PD. Importantly, h-FTAA detected aggregation events at an earlier stage than ThT, underscoring its suitability for capturing transient intermediates that may otherwise evade detection.

In cellular models, stable expression of huWT- or huA53T-αsyn in HEK293 cells provided a physiologically relevant context for investigating seeded aggregation. Exposure to preformed fibrils successfully induced intracellular inclusions, with huA53T-seeded cells exhibiting more prominent and earlier aggregation phenotypes. Immunocytochemical labeling with pS129-αsyn corroborated these observations, while spectral emission and fluorescence lifetime imaging of h-FTAA revealed subtle but consistent differences in aggregate microenvironments between WT and A53T contexts. Emission profiles in cells were red-shifted and broadened compared to in vitro fibrils, indicating heterogeneous binding to multiple conformers. Fluorescence lifetimes were consistently shorter in cellular contexts (0.68–0.87 ns vs. ~1 ns in vitro), with variations over time (days 7 to 9 post-exposure) and between genotypes, reflecting dynamic molecular milieus—possibly pH, polarity, or subcellular compartments—affecting aggregate morphology. It was not possible to resolve any distinct stages of the fibrillation process in the cell models using h-FTAA, probably because of the heterogeneous local nature of the aggregation process itself. Together, these findings establish two key conclusions. First, h-FTAA is an effective and sensitive reporter for distinguishing αsyn aggregate polymorphs across different cellular and biochemical environments. Second, the intrinsic cellular environment plays an active role in shaping αsyn aggregation, influencing not only the kinetics but also the structural conformations that emerge. This has significant implications for synucleinopathy research, as disease progression may not only depend on intrinsic αsyn mutations but also on cell-type-specific environments.

By providing both an in vitro and cellular framework for studying early αsyn morphotypes, this work advances the methodological toolkit available for probing synucleinopathies. Future research should extend these approaches to neuronal systems and patient-derived models to further clarify how early aggregation signatures can serve as biomarkers or therapeutic targets. Ultimately, the ability to resolve and track distinct αsyn conformers in their native environments offers a promising path toward earlier diagnosis and more precise interventions in PD and related disorders.

## Data Availability

Further detailed data are included in the Supplementary Information connected to this publication.

## Supplementary Materials

The following supporting information can be downloaded at https://www.mdpi.com/article/10.3390/cells14191542/s1, Figure S1: Original Western blots.; Figures S2–S7: Additional fluorescence microscope images. Figure S8: Additional Emission spectra and FLIM data. Figures S9 and S10: Additional FLIM images.
