# Supplementary material for: Spectral Profiling of Early αsyn Aggregation in HEK293 Cells Modified to Stably Express Human WT and A53T-αsyn
Source: Cells. 2025 Oct 2;14(19):1542. doi: 10.3390/cells14191542 (PMC12523747; doi:10.3390/cells14191542)
Supplement: Supplementary file 1 [file cells-14-01542-s001.zip › cells-3873741-supplementary.pdf]

Supplementary information: Spectral profiling of early αsyn aggregation in HEK293 cells modified to stably express human WT and A53T αsyn

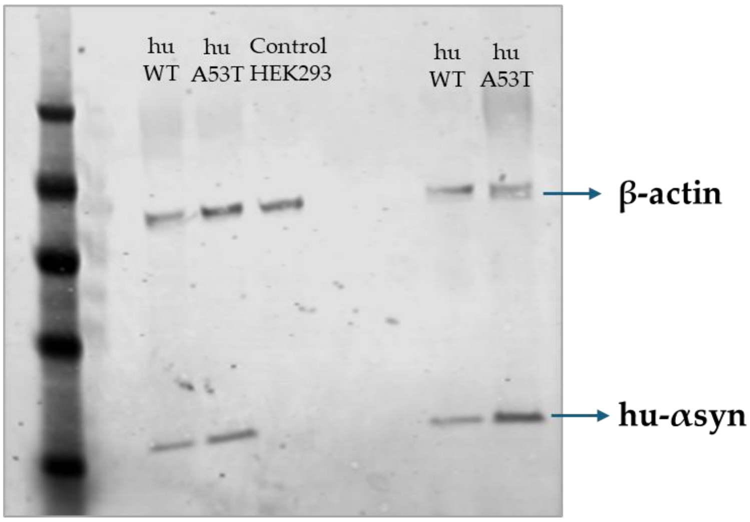

Figure S1: Original western blotting image showing endogenous hu-αsyn expression in HEK 293 cells, transfected with 4µg of huWT or huA53T-αsyn containing pcDNA 3.1 plasmid. αsyn protein bands were detected at ~15kDa in stable HEK293 cells whereas no bands were seen in control HEK293. B-actin was added as a loading control which was detected ~42kDa in all the cell types.

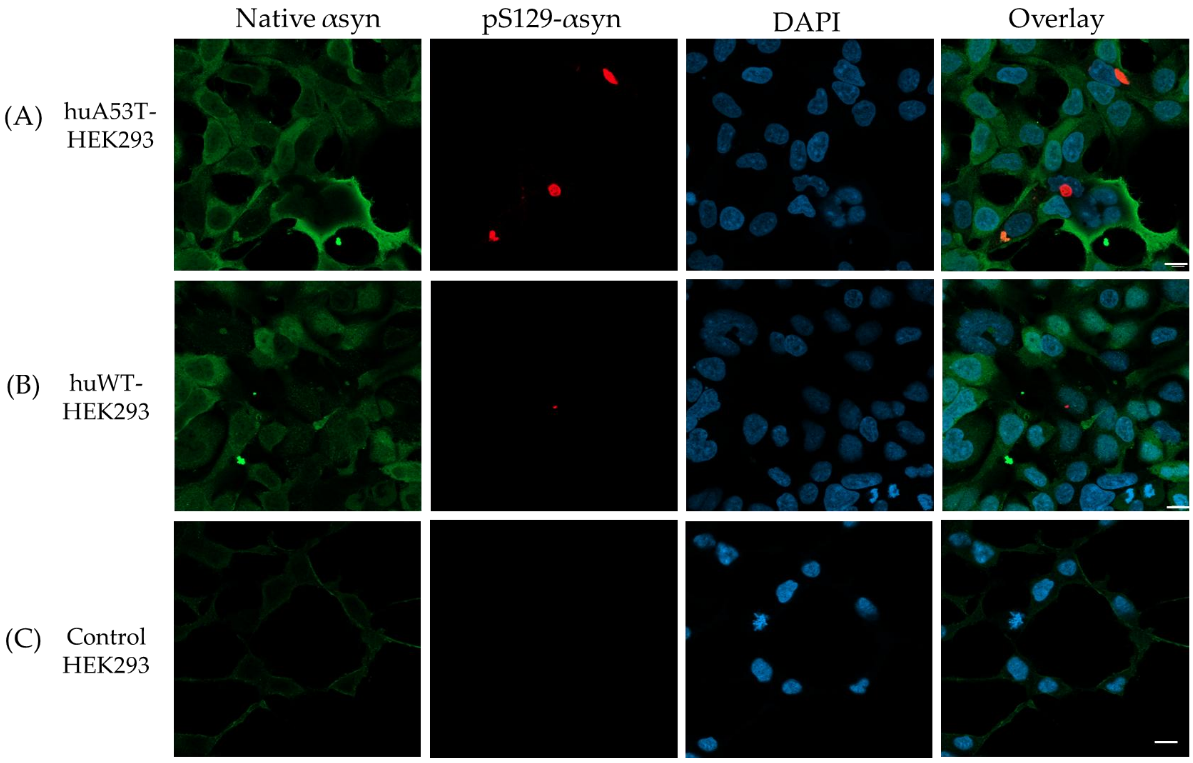

Figure S2: Representative fluorescence images of (A) huA53T-HEK293 and (B) huWT-HEK293 cells, exposed to 1μM huWT-αsyn PFFs and labelled with Syn211 (green, 1:500) + pS129-αsyn (red, 1:2000). (C) Control HEK293 cells, also transfected with huWT-αsyn PFFs, exhibit minimal background from Syn211 + pS129-αsyn. Images were acquired on day 9 following initial PFFs exposure, at 40X magnification. Cell nuclei were stained with 1μg/ml DAPI (blue). Scale bar represents 10μm.

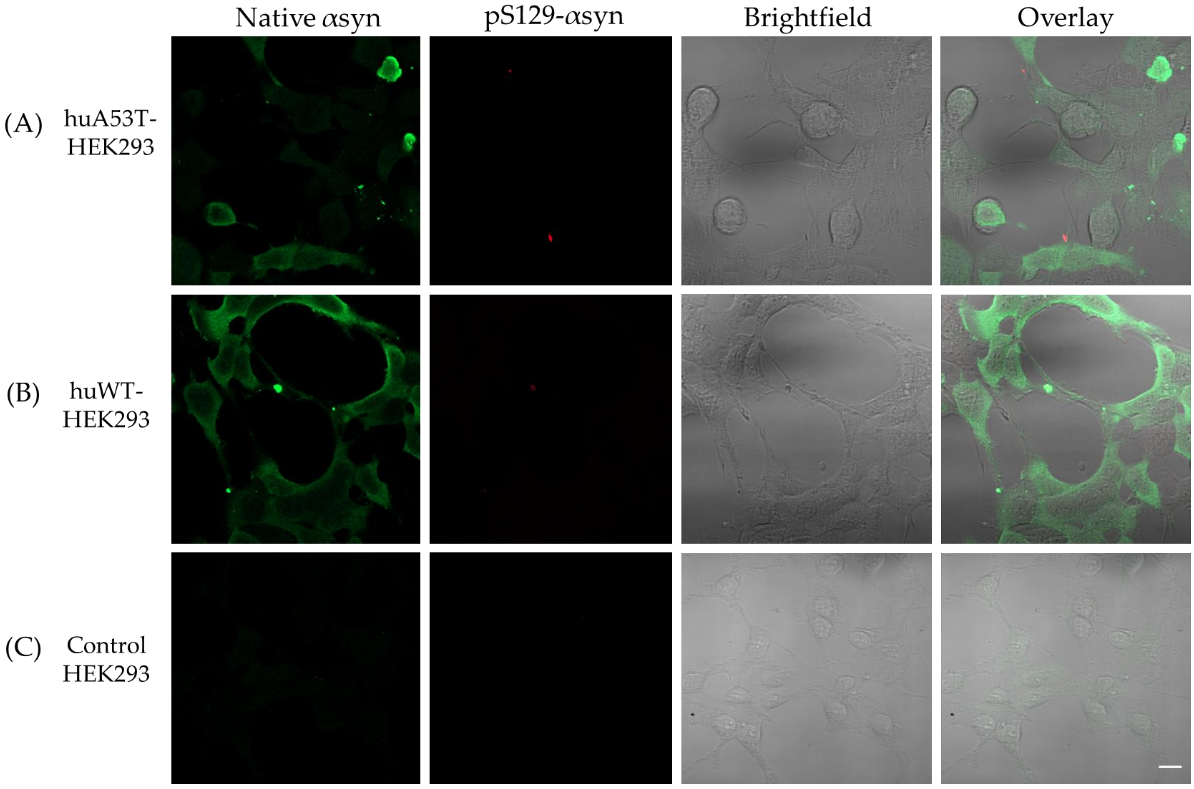

Figure S3: Representative fluorescence and brightfield images of (A) huA53T-HEK293, (B) huWT-HEK293 cells and (C) control HEK293 cells, exposed to 1uM huA53T-αsyn PFFs, counterstained with Syn211 (green, 1:500) + pS129-αsyn (red, 1:2000). (1µg/ml). Images were acquired on day 9 post initial fibril seeding. The scale represents 10µm, 40X objective used.

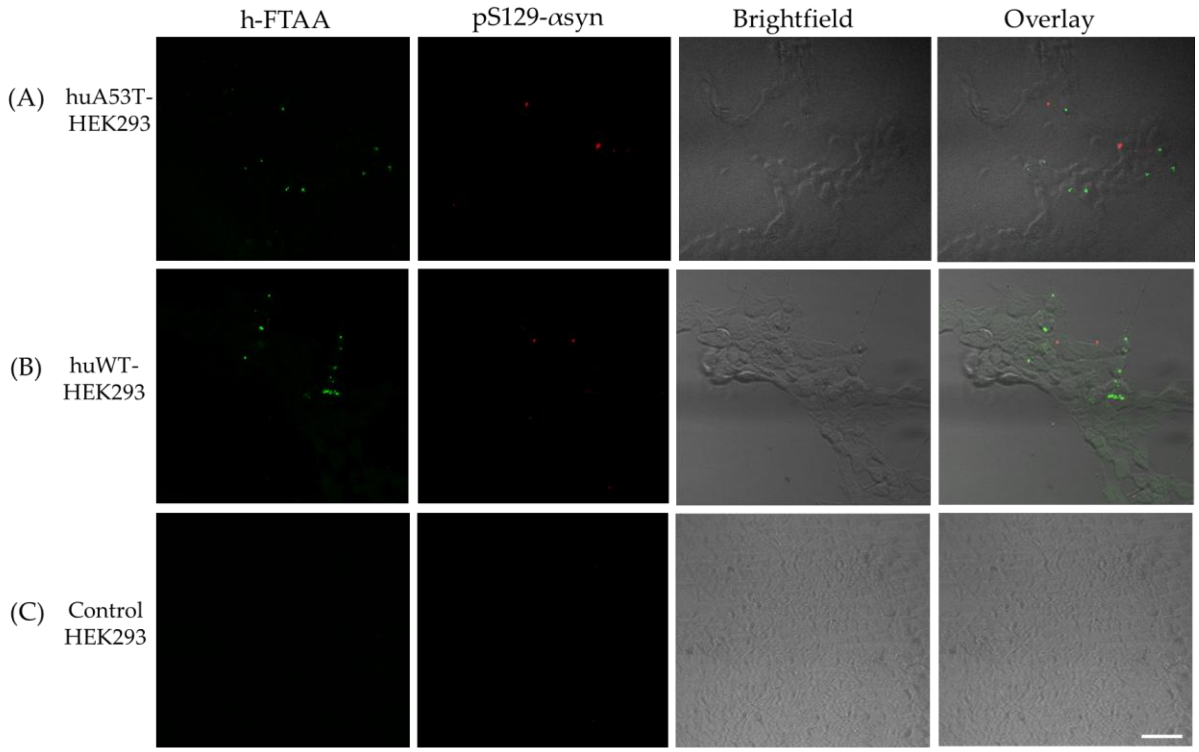

Figure S4: Representative fluorescence and brightfield images of (A) huA53T-HEK293, (B) huWT-HEK293 and (C) Control HEK293 cells exposed to 1 μM huWT-αsyn PFFs, co-labelled with 1uM h-FTAA (green) and pS129-αsyn antibody (red, 1:2000). Images were captured on day 7 following fibril seeding, using 20X objective. The scale bar represents 50 μm.

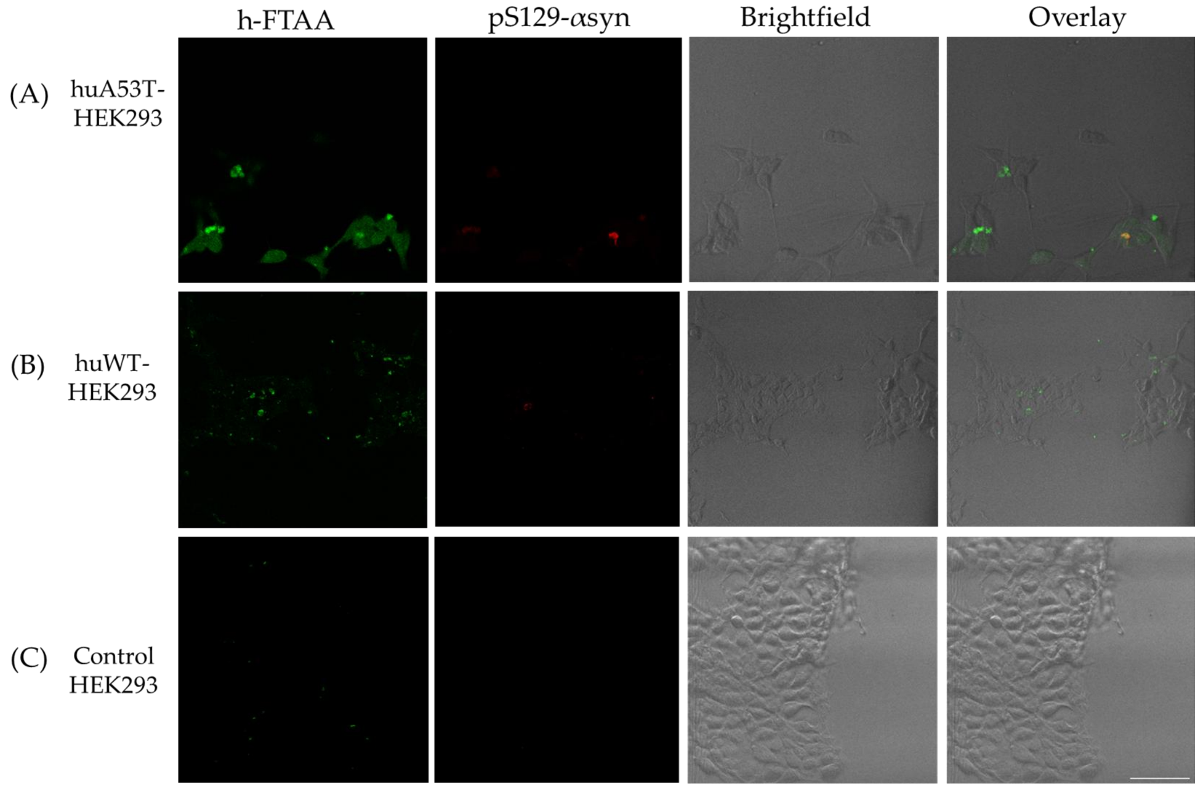

Figure S5: Representative fluorescence and brightfield images of (A) huA53T-HEK293, (B) huWT-HEK293 and (C) Control HEK293 cells exposed to 1 μM huA53T-αsyn PFFs, stained with 1μM h-FTAA (green) + pS129-αsyn antibody (red, 1:2000). Images were acquired using 20X objective, on day 7 following fibril exposure. The scale bar represents 75 μm.

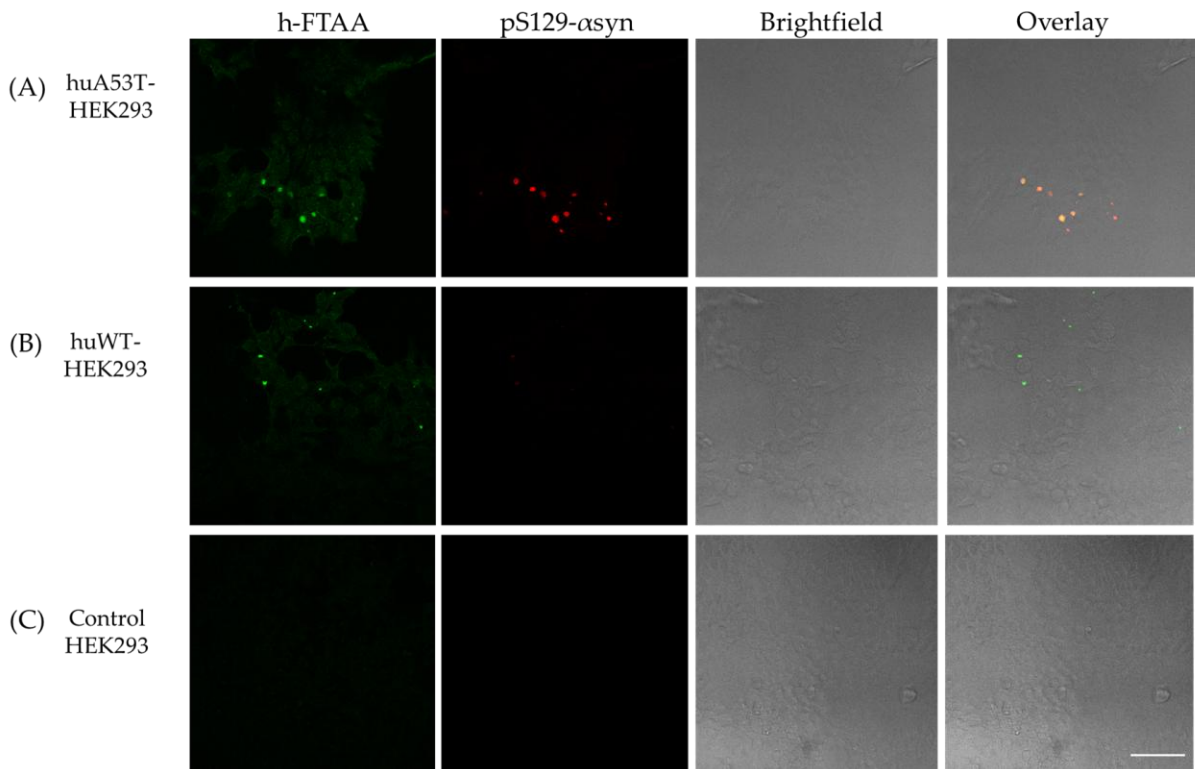

Figure S6: Representative fluorescence and brightfield images of (A) huA53T-HEK293, (B) huWT-HEK293 and (C) Control HEK293 cells exposed to 1μM huWT-αsyn PFFs, stained with 1μM h-FTAA (green) + pS129-αsyn antibody (red, 1:2000). Images were acquired using 20X objective, on day 9 following fibril exposure. The scale bar represents 50 μm.

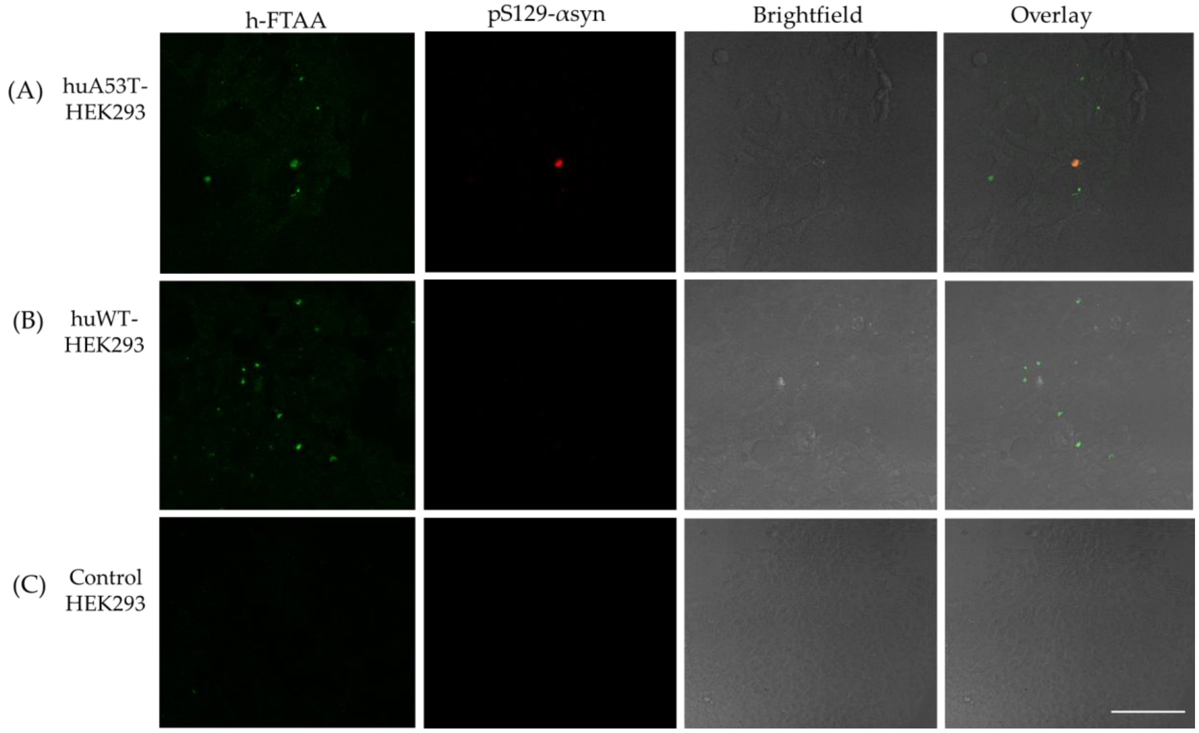

Figure S7: Representative fluorescence and brightfield images of (A) huA53T-HEK293, (B) huWT-HEK293 and (C) Control HEK293 cells exposed to 1μM huA53T-αsyn PFFs, stained with 1μM h-FTAA (green) + pS129-αsyn antibody (red, 1:2000). Images were acquired using 20X objective, on day 9 following fibril exposure. The scale bar represents 75μm.

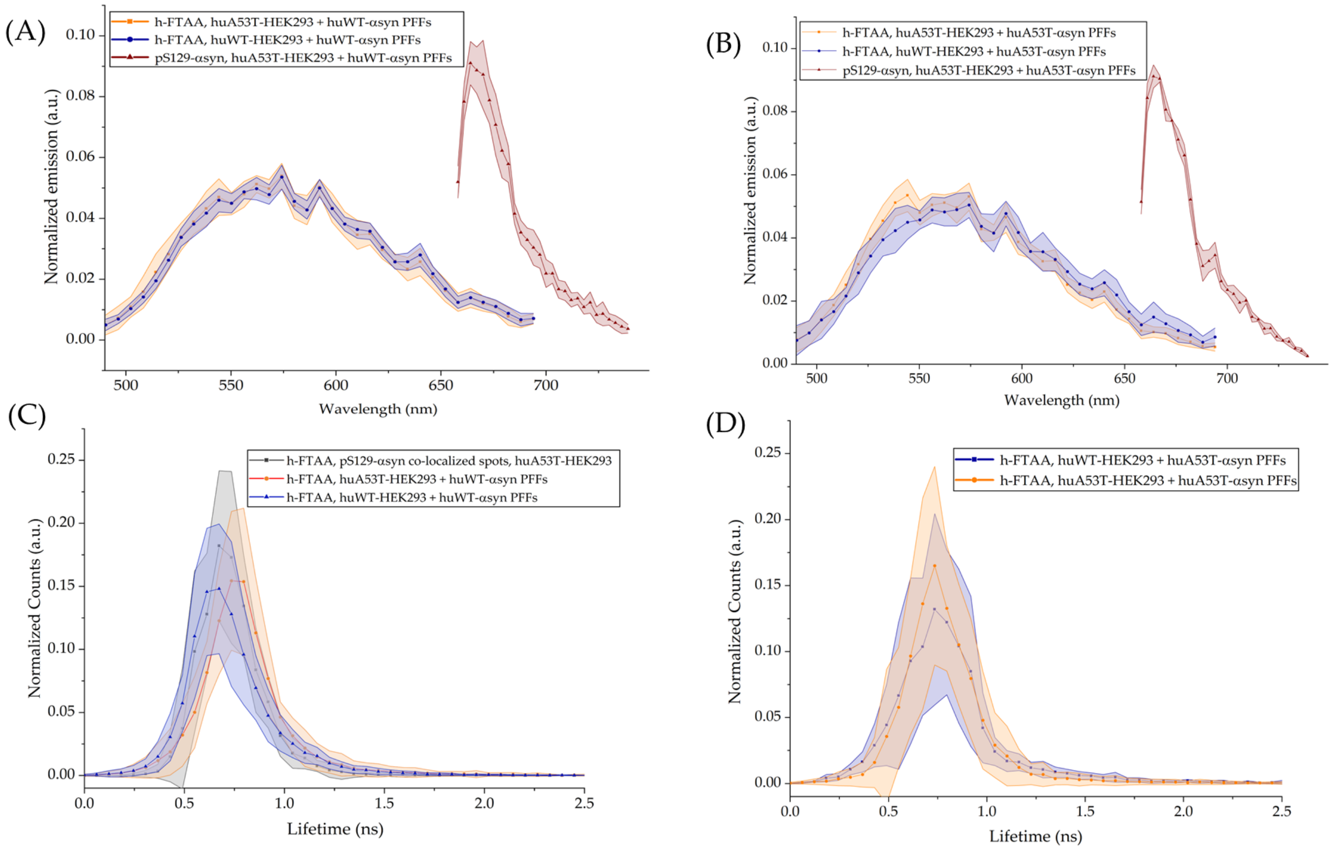

Figure S8: Spectral emission profiles and fluorescence lifetime distributions of h-FTAA (also includes h-FTAA-labelled, pS129-αsyn positive puncta), bound to aggregates in huWT- and huA53T-HEK293 cells when exposed to A), C) 1μM huWT- or B), D) huA53T-αsyn PFFs. These plots correspond to the fluorescence and FLIM images taken on day 9, following initial exposure to fibrils. The λ_{ex} for h-FTAA was at 475 nm and λ_{em} was measured using 490 - 695 nm range. The λ_{ex} for Alexa fluor 647 (pS129-αsyn probing) was at 650nm and λ_{em} was recorded using 658 - 745 nm range.

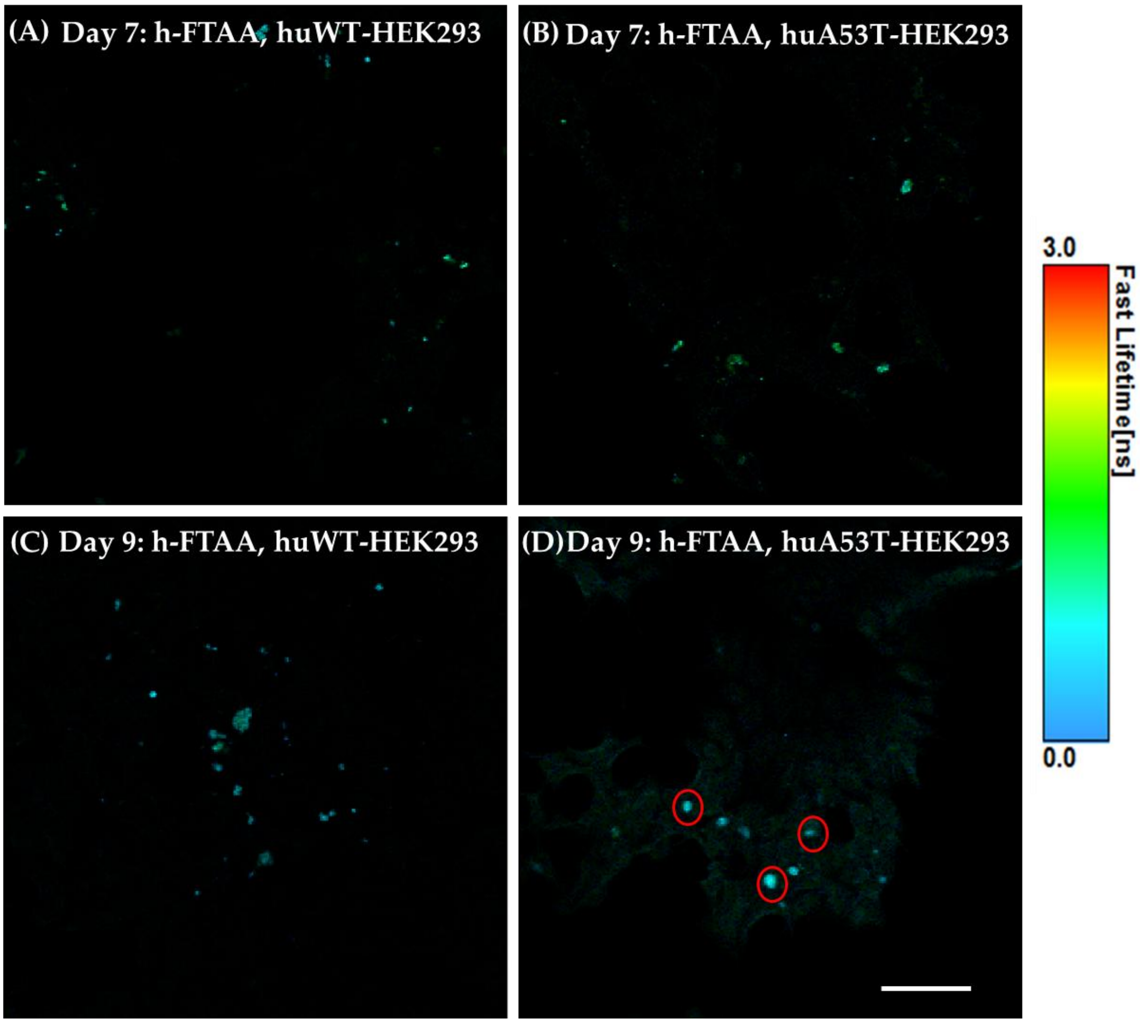

Figure S9: Representative FLIM images of h-FTAA-probed intracellular aggregates, when stable HEK293 cells were exposed to 1μM huWT-αsyn PFFs. FLIM images of (A) huWT-HEK293, (B) huA53T-HEK293 cells were recorded on day 7 and (C) huWT-HEK293, (D) huA53T-HEK293 cells were recorded on day 9 following initial PFFs exposure. The h-FTAA labelled, pS129-positive puncta within the cells are marked with red circles. The h-FTAA was excited at 475nm, and the emission was recorded using 490- 695 nm range. The color bar represents lifetime ranging from 0 to 3 ns. The scale bar represents 40μm.

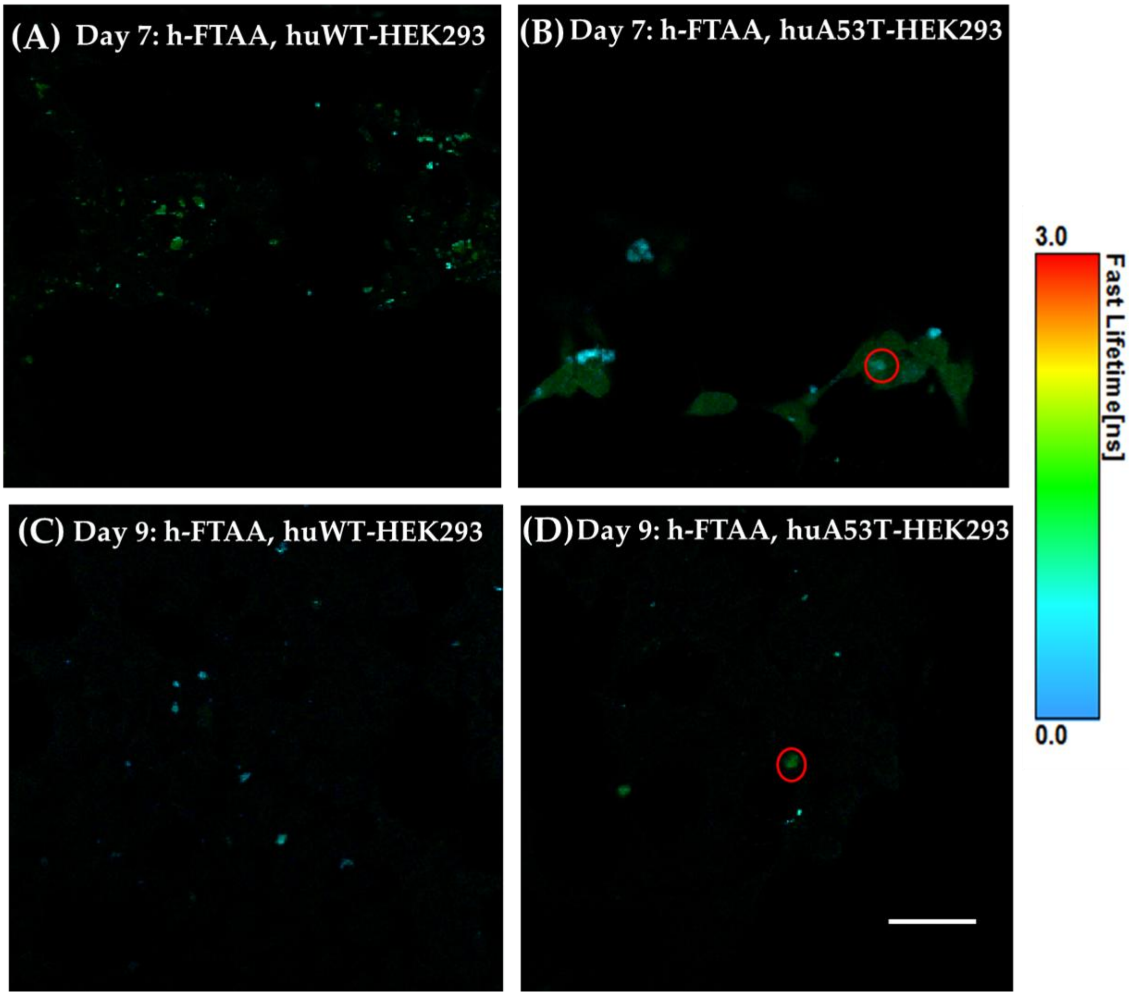

Figure S10: Representative FLIM images of h-FTAA-probed intracellular aggregates, when stable HEK293 cells were exposed to 1μM huA53T-αsyn PFFs. FLIM images of (A) huWT-HEK293, (B) huA53T-HEK293 cells were recorded on day 7 and (C) huWT-HEK293, (D) huA53T-HEK293 cells were recorded on day 9 following initial PFFs exposure. The h-FTAA labelled, pS129-positive puncta within the cells are marked with red circles. The h-FTAA was excited at 475nm, and the emission was recorded using 490- 695 nm range. The color bar represents lifetime ranging from 0 to 3 ns. The scale bar represents 40μm.
